# The Olympic Biathlon – Recent Advances and Perspectives After Pyeongchang

**DOI:** 10.3389/fphys.2018.00796

**Published:** 2018-07-02

**Authors:** Marko S. Laaksonen, Malin Jonsson, Hans-Christer Holmberg

**Affiliations:** ^1^Swedish Winter Sports Research Centre, Department of Health Sciences, Mid Sweden University, Östersund, Sweden; ^2^School of Sports Sciences, UiT The Arctic University of Norway, Tromsø, Norway; ^3^School of Kinesiology, The University of British Columbia, Vancouver, BC, Canada

**Keywords:** performance, physiology, shooting, skiing, training

## Abstract

The biathlon, combining cross-country ski skating with rifle marksmanship, has been an Olympic event since the Winter Games in Squaw Valley, United States, in 1960. As a consequence of replacing the classical with the skating technique in the 1980s, as well as considerable improvements in equipment and preparation of ski tracks and more effective training, the average biathlon skiing speed has increased substantially. Moreover, the mass-start, pursuit, and sprint races have been introduced. Indeed, two of the four current individual Olympic biathlon competitions involve mass-starts, where tactics play a major role and the outcome is often decided during the last round of shooting or final sprint. Biathlon is a demanding endurance sport requiring extensive aerobic capacity. The wide range of speeds and slopes involved requires biathletes to alternate continuously between and adapt different skating sub-techniques during races, a technical complexity that places a premium on efficiency. Although the relative amounts of endurance training at different levels of intensity have remained essentially constant during recent decades, today’s biathletes perform more specific endurance training on roller skis on terrain similar to that used for competition, with more focus on the upper-body, systematic strength and power training and skiing at higher speeds. Success in the biathlon also requires accurate and rapid shooting while simultaneously recovering from high-intensity skiing. Many different factors, including body sway, triggering behavior, and even psychology, influence the shooting performance. Thus, the complexity of biathlon deserves a greater research focus on areas such as race tactics, skating techniques, or shooting process.

## Introduction

The biathlon, an Olympic sport that combines rifle marksmanship and cross-country (XC) skiing with the skating technique while carrying a rifle, involves considerable physiological demands similar to those associated with competitive XC skiing (Hoffman and Street, 1992; Sandbakk and Holmberg, 2014; Holmberg, 2015), while also requiring precise fine motor control for fast and accurate shooting under mental pressure (Vickers and Williams, 2007). Moreover, this challenging endurance sport entails alternating between various sub-techniques that require different relative amounts of upper- and/or lower-body work while skiing on varying terrain. This necessitates extensive training designed not only to optimize the relevant physiological capacities and performance of various ski skating techniques, but also to improve and maintain accurate shooting within a short period of time.

### The Evolution of Olympic Biathlon Competition

The biathlon first became an Olympic event in the Winter Games in Squaw Valley, United States, in 1960. The development of the skating technique in the 1980s (Smith, 1990), in combination with substantial improvements in equipment, track preparation, and training, has increased the average skiing speeds in biathlon races considerably (IBU, 2018). Moreover, new events such as the sprint, pursuit, and mass-start have been introduced.

The recent Olympic Games in Pyeongchang in 2018 involved six types of biathlon races (**Table 1**), three of which were added after the Olympic Games in Nagano in 1998 – the pursuit competitions in 2002 at Salt Lake City, the mass-start in 2006 at Turin, and the mixed relay in 2014 at Sochi.

**Table 1:** The different types of biathlon competition at the Pyeongchang Olympic Games in 2018.

Competition	Type of start	Skiing distance (km)	Shooting	Competition time (mm:ss)	Skiing time (mm:ss)	Range time (mm:ss)	Shooting time (mm:ss)	Type of penalty	Duration on the penalty loop (s)
Sprint	Single-start	W: 7.5 (3 × 2.5)	P+S	W: 20:00–23:00	W: 18:00–20:30	W: 1:30–2:00	W: 0:50–1:10	Penalty loop	W: 23.0–26.0
		M: 10 (3 × 3.3)		M: 22:30–26:30	M: 21:00–24:30	M: 1:20–1:50	M: 0:50–1:05	(150 m)	M: 19.5–22.5
Pursuit	Mass-start based on results of the sprint	W: 10 (5 × 2)	P+P+S+S	W: 30:00:34:00	W: 25:30–29:30	W: 3:10–3:40	W: 1:45–2:15	Penalty loop	W: 23.0–26.0


		M: 12.5 (5 × 2.5)		M: 31:00–33:30	M: 26:30–29:00	M: 2:50–3:15	M: 1:40–2:10	(150 m)	M: 19.5–22.5
Mass-start	Mass-start	W: 12.5 (5 × 2.5)	P+P+S+S	W: 34:00–37:30	W: 30:00–33:30	W: 3:10–3:30	W: 1:45–2:10	Penalty loop	W: 23.0–26.0
		M: 15 (5 × 3)		M: 36:00–38:30	M: 32:00–34:00	M: 2:45–3:10	M: 1:35–2:00	(150 m)	M:19.5–22.5
Individual	Single-start	W: 15 (5 × 3)	P+S+P+S	W: 41:30–43:00	W: 37:30–39:00	W: 3:15–3:45	W: 1:50–2:20	Penalty time	NA
		M: 20 (5 × 4)		M: 44:30–48:00	M: 40:30–44:30	M: 3:00–3:30	M: 1:45–2:15	(1 min)	
Relay	Mass-start	W: 4 × 6 (3 × 2)	4 × P+S	W: 4 × 17:30–19:00	W: 4 × 15:00–16:30	W: 4 × 1:30–2:30	W: 4 × 0:50–2:00	Penalty loop	W: 23.0–26.0
		M: 4 × 7.5 (3 × 2.5)		M: 4 × 18:30–20:30	M: 4 × 16:30–18:00	M: 4 × 1:30–2:30	M: 4 × 0:50–2:00	(150 m)	M: 19.5–22.5
Mixed Relay	Mass-start	W: 2 × 6 (3 × 2) +	4 × P+S	W: 2 × 16:00–18:30 +	W: 2 × 14:00–16:30 +	W: 4 × 1:30–2:15	W: 4 × 0:50–1:30	Penalty loop	W: 23.0–26.0
		M: 2 × 7.5 (3 × 2.5)		M: 2 × 18:00–19:30	M: 2 × 16:00–17:30	M: 4 × 1:30–2:15	M: 4 × 0:50–1:30	(150 m)	M:19.5–22.5


W, women; M, men; P, prone position; S, standing position; NA, not applicable.

## The Demands of Olympic Biathlon Competition

Although the duration of biathlon races ranges from 20 min (the sprint) to more than 50 min (the individual race), seven of the 11 Olympic events (including relays) involve mass-starts, which enhance the importance of tactics and where the outcome is often decided by the last round of shooting and/or the final skiing sprint. The overall performance in the biathlon is complex, being decided by several components, such as skiing speed, range time (time spent on the shooting ramp), shooting time, and shooting accuracy. Usually, the range and shooting times of elite biathletes and in different types of competitions are similar and, thus, exert only a minor impact on the final performance. In contrast, skiing speed and shooting accuracy are the most important determinants of the final outcome (Skattebo and Losnegard, 2018).

### Skiing

The biathlon race courses are required to consist of continuously changing flat, uphill, and downhill sections (IBU, 2017), forcing frequent alternation between the various skating sub-techniques (Holmberg, 2015). The demands of biathlon skiing are comparable to those made by XC skiing, where more than 50% of the racing time is spent on uphill terrain, the sections on which individual performance varies most (Bergh and Forsberg, 2000; Andersson et al., 2010; IBU, 2018). World-class male and female biathletes demonstrate high maximal oxygen uptakes (VO_2_max) of >80 and >65 mL⋅kg^-1^⋅min^-1^, respectively (Tønnessen et al., 2015). The best competitors are well-trained endurance athletes, excellent at skiing with the skating technique, and, in several cases, also able to compete at a high level in elite XC ski races.

In addition to adapting their speed to the track profile, snow conditions, and altitude, biathletes (in contrast to XC skiers) must prepare for the coming shooting. Thus, unlike XC skiing, biathlon skiing is intermittent, being interrupted by short stops on the shooting range. It has been proposed that that skiing speed is responsible for more than 60% of the overall performance in World Cup biathlon sprint competitions (Luchsinger et al., 2018), but it is currently unknown how the skiing speed influences the overall performance in different types of biathlon competitions or on different terrains. It is to be expected that in connection with pursuit and mass-start races, the skiing speed exerts less impact on the overall performance than in sprint, since the former events involve four bouts of shooting with shorter skiing loops between. During mass-start races, drafting behind other skiers, locating oneself optimally in the crowd also helps maximize the utilization of individual strengths.

From a biomechanical perspective, biathletes resemble XC skiers, employing a wide range of speeds over varying terrain with continuous transitions between the different sub-techniques (Andersson et al., 2010), also called gears^1^, as well as utilizing the tuck position and several different turning techniques downhill (Sandbakk Ø. et al., 2014; Sandbakk S.B. et al., 2014). These many transitions between gears require not only mastery of the various skating sub-techniques, but also effective timing. Ski skating at the racing speed requires both long and rapid cycles (Stöggl and Müller, 2009; Stöggl et al., 2011; Sandbakk et al., 2012), with length being especially important on flat terrain and rapid cycles, with as little reduction in cycle length as possible, on steep uphill terrain and during the final sprint. The technical complexity involved, with numerous possibilities for timing the generation of force by the arms and legs, offers both opportunities and presents challenges. To date, few investigations have considered how carrying a rifle (minimum weight 3.5 kg) while skiing influences energy cost/skiing physiology and biomechanics (Rundell and Szmedra, 1998; Stöggl et al., 2015), as well as the choice of sub-technique.

### Shooting

Overall, the shooting accuracies in the prone and standing positions are comparable, probably because of the difference in the diameter of the target hit areas (4.5 versus 11.5 cm, respectively; IBU, 2018). In the Sochi Olympic Games in 2014, the average shooting accuracy for all individual male and female medalists was 97%. Under the more difficult wind conditions encountered in the recent Pyeongchang Games, the corresponding values were 93 and 95%, respectively. However, this level of accuracy is actually greater than the long-term accuracy of these same athletes, indicating a high degree of randomness in connection with biathlon shooting (Maier et al., 2018). Altogether, if a biathlete hopes to win an Olympic medal under normal weather conditions, he/she cannot miss even once during the two rounds of shooting in sprint races and incur no more than one penalty in connection with the four shootings in the other individual events.

During the 15–30 s prior to shooting, the biathlete slows down slightly, with the duration of this slowdown being highly individual and dependent on the terrain. After stopping in the shooting lane, the biathlete takes the position and fires the first shot within 15 s, the entire series of five shots lasting approximately 10 s. During this time, the heart rate usually falls from approximately 90 to 60 or 70% of HR_max_ during prone or standing shooting, respectively (Hoffman and Street, 1992). However, the intensity of exercise prior to shooting has been proposed to have only a minimal impact on the shooting performance (Hoffman et al., 1992).

The necessity to prepare, fire five shots, and exit the shooting lane within approximately 25–30 s is highly stressful. However, the time spent on the shooting range and shooting time varies relatively little between elite biathletes and, therefore, contributes to the overall performance to only a minor extent (∼2–4%; Luchsinger et al., 2018; Skattebo and Losnegard, 2018). At the same time, approximately 35% of the overall biathlon sprint performance is determined by shooting accuracy (Luchsinger et al., 2018), a value that may be as high as 50% in connection with individual biathlon races, where each missed shot results in a 1-min penalty.

Several aspects of the shooting technique influence the performance. In the prone position, the triggering behavior and rifle sway of world-class biathletes distinguish them from lower level competitors, and rifle sway is also an important factor in the standing position (Sattlecker et al., 2017). The preceding exercise almost certainly influences the psychophysiological aspects of the arousal/activation and focused attention required to perform the complex task of aiming successfully, since the several tasks (aiming, maintaining optimal body posture, and triggering) performed simultaneously demand extensive fine-motor control. A more in-depth systematic analysis of the biomechanics of shooting in both the prone and standing positions under pronounced stress needs to be performed.

Weather conditions, and especially wind, exert a considerable impact on the shooting strategy. Although the wind speed appears to exert only a minor effect on the overall shooting accuracy (Skattebo and Losnegard, 2018), the wind must be taken into consideration and sometimes the biathlete must wait until the wind subsides. In addition, when shooting in standing position, depending on the layout of the stadium, it can be beneficial to shoot from lanes where the wind is lighter and affects body sway less. Thus, future studies in biathlon shooting should also assess the effects of weather conditions, including temperature, wind (especially speed and direction), and visibility (snowfall and fog).

## Training for Olympic Biathlon Races

The best biathletes perform 700–900 h of physical training annually, including endurance training of approximately 80% at low, 4–5% at moderate, and 5–6% at high intensity, together with 10% of strength and speed training (**Table 2**; personal communication with Swedish biathlon coaches). This volume of training is slightly less than that reported earlier for XC skiers (Sandbakk and Holmberg, 2014), probably due to the time spent on training shooting. Usually, exercise intensities are chosen on the basis of laboratory testing and approximately 60–70% of the annual training is performed from May to November and the remainder during the competitive season from December to April. The season starts with more low-intensity training and the relative portions of moderate- and high-intensity training increase as the training season progresses. Roller skiing, cycling, and running on varying terrain are the predominant modes from May to October, with only a few days of training on snow each month, whereas from November onward, most training involves skiing on snow. The main technique is skating, with classical skiing being performed only during long sessions at low intensity or for recovery.

**Table 2:** Overview of training by the successful Swedish biathletes who won medals at the Pyeongchang Olympic Games in 2018.

Physical training	Shooting training
In total, 700–900 h of endurance training	In total, ∼22 000 shots fired during ∼210 sessions
550–700 h training at low intensity (60–80 % HR_max_)	∼7000 shots at rest (during ∼45 sessions from May to the middle of August)
30–45 h training at moderate intensity (80-90 % HR_max_)	2400 shots for training precision (∼20 sessions)
35–50 h training at high intensity, including races (>90% HR_max_)	2400 shots for training under stress (∼24 sessions)
10–15 sessions of anaerobic lactic acid training	120–130 sessions ”dry shooting”
10–15 sessions of speed/power training	2000–3000 shots to zero the rifle (training and competition combined)
40–50 sessions of maximal or explosive strength training	∼12 000 shots in combination with physical training [A1–A3 (roller-)skiing, running]
40–45 sessions of body stability/muscle activation strength training	∼700 shots during competitions


### Distribution of the Intensity of Training

Low-intensity training has been proposed to enhance overall aerobic capacity and exercise efficiency, as well as to improve “tolerance” for high training loads by accelerating recovery (Tønnessen et al., 2014). Although most low-intensity training is designed to develop aerobic capacity and/or specific motor skills, the inclusion of some semi- or un-specific training (e.g., cross-training) allows more overall exercise to be performed.

Training at moderate intensity (i.e., directly below the anaerobic threshold) can be prolonged while maintaining an adequate supply of aerobic energy. Such sessions commonly include long intervals of exercise, interspersed with short periods of recovery, or continuous exercise for 30–60 min. To control the intensity, such training is carried out preferably on a relatively constant terrain. Moderate-intensity training is performed once or twice a week during the period of preparation and less often during the competitive season.

Although the best athletes focus on extensive low-intensity training, the beneficial effects of high-intensity training on endurance performance have been demonstrated repeatedly (Laursen and Jenkins, 2002). At the same time, there is increasing awareness that highly trained athletes should focus more on improving the quality of each high-intensity session (i.e., optimization of physical, technical, and mental aspects) than on the number of such sessions.

### The Mode of Exercise

While their high-intensity training involves pre-dominantly roller-skiing and skiing, biathletes vary their low-intensity exercise considerably. During the 6 months of preparation, a biathlete gold medalist devotes 50–60% of his/her time to sport-specific training and most of the remainder to cycling and, to lesser extent, running (M. Laaksonen, personal communication, March 21, 2018). Biathletes presumably cycle more than XC skiers, since the former employs only the skating technique, which activates the legs (thighs) more extensively. While training (roller-) skiing in combination with shooting, biathletes may also carry a rifle on their back, but this is done surprisingly little (15–20% of all endurance training) and, thus, offers a considerable opportunity for future development.

In addition, biathletes must perform various skating techniques, which load the upper and lower body to different extents, efficiently. The choice of sub-technique is influenced by the speed and external conditions (e.g., the profile of the terrain, snow conditions, waxing of skis, and altitude), as well as the individual level of performance and physical characteristics. For example, since 50% of racing time is spent skiing uphill, training these sub-techniques is especially important. Overall, biathletes must be aware not only of the mode and intensity of their exercise, but also of how they train the arms, legs, and entire body.

### Training Speed and Strength

The increase in biathlon skiing speed during the last 20 years has involved an enhanced development of speed and strength. Thus, both male and female world-class biathletes train skiing speed, often in sessions involving 10–20 sprints at maximal intensity (depending on technique and terrain) with 2–3-min intervals of recovery.

However, to date, the effects of strength training on biathlon performance have not been documented. Several studies on XC skiers have revealed that movement-specific training of maximal upper body strength improves, in particular, double poling (Nilsson et al., 2004), although this technique is not used on its own in the biathlon. Overall, available findings allow us to speculate that for biathletes who train endurance extensively, additional training of strength and speed could develop and maintain muscle mass and power, particularly in the case of the upper body of women (Holmberg, 2005; Hegge et al., 2016), as well as improve the skating technique while carrying the rifle. However, the potential effects of combined speed and endurance training require considerably more evaluation.

### Training Shooting

The shooting time between bouts of skiing, usually 25–30 s in both the prone and standing positions, includes preparation (10–15 s for taking position), shooting (10–15 s for aiming and firing five shots), and exit (3–5 s). During a single season, world-class biathletes fire more than 20,000 shots during more than 200 training sessions, approximately 60% of which involve shooting combined with endurance training [9,000 (75%) at low, 2,000 (15%) at moderate, and 1,250 (10%) at high intensity], i.e., shooting between bouts of skiing or, to lesser extent, running (**Table 2**). Although the basics of such training have not changed significantly in recent decades, the shooting time and accuracy have tended to improve, emphasizing the importance of training under conditions that resemble those in a competition (e.g., biathlete against biathlete or under time pressure).

The remainder of these more than 20,000 shots are fired at rest, focusing on improving the accuracy and/or the speed of preparation, shooting, and exit. Indeed, many world-class biathletes now focus especially on preparing rapidly for the first shot and leaving the shooting lane as quickly as possible. Shooting at rest as well as shooting without ammunition (so-called dry shooting) can also improve triggering behavior, rifle stability, and/or holding (Groslambert et al., 2003), as well as mental aspects of shooting (Laaksonen et al., 2011). Thus, training under conditions that resemble competitive shooting is recommended for elite biathletes, not only to improve the accuracy but also to minimize the loss of time on the range and while shooting. Usually, preparation begins with shooting at rest (May), later progressing to shooting in connection with endurance training (June to November).

Outdoor conditions also influence the accuracy of biathlon shooting. Accordingly, training under windy conditions is recommended, since when shooting in the standing position rifle stability is strongly correlated to scores (Groslambert et al., 1999) and discriminates low- from high-scoring biathletes (Sattlecker et al., 2017). Moreover, rifle motion and body sway are related (Ihalainen et al., 2018), the latter being less pronounced in elite athletes (Niinimaa and McAvoy, 1983) and also clearly distinguishing low- from high-level shooters (Groslambert et al., 1999). Thus, balance training in connection with shooting is also beneficial for biathletes, as in the case of rifle shooters (Era et al., 1996).

In addition, trigger force prior to firing discriminates between elite and young athletes shooting while standing (Sattlecker et al., 2009). In general, exercise prior to shooting lessens this trigger force, but interestingly, elite biathletes are capable of maintaining their high pre-shot trigger force at rest even immediately after exercise (Sattlecker et al., 2013).

## Future Perspectives

The current Olympic biathlon program will not be changed in connection with the next several Olympic Games, so the associated demands will probably not change as much as in previous years. However, since the skiing speed and shooting time of Olympic medalists are relatively similar, the shooting accuracy better than the biathlete’s long-term average will become even more important in the future. The physiology and biomechanics of biathletes have been investigated much less extensively than those of XC skiers and, moreover, relatively little is known about actual competitions.

Recent advances in sensor technology allow the position, speed, kinematics, and kinetics of biathletes to be recorded in real time on the track, providing more detailed information concerning the determinants of success in the Olympic Games. Furthermore, the enhanced complexity of both physiological (unaltered aerobic, but more pronounced anaerobic demands) and technical training (a large number of sub-techniques to master, improved shooting technique) by modern biathletes accentuates inter-individual variations in adaptation and response. In addition, pacing strategies in different types of biathlon races, as well as the potential effects of pacing and exercise on shooting performance require examination. Furthermore, more comprehensive analysis of the shooting process, especially during actual competition, will help formulate training guidelines for future Olympic champions. However, the need to increase biathlon-specific training while carrying a rifle and to match training precisely to the unique characteristics of each individual biathlete will continue to challenge researchers for years to come.

Although much of the extensive literature on XC skiing and rifle shooting may be relevant to the biathlon, carrying a rifle while skiing and shooting under physiological and psychological stress are somewhat unique features. The number of publications about XC skiing is approaching 700 (April 19, 2018), while those on the biathlon have risen from 29 in 2006 (the Olympic Games in Turin) to almost 80 in 2018 (the Olympics Games in Pyeongchang). Although much of this latter research has focused on shooting and medical aspects, the last decade has seen a clear trend toward more interest in physiology and biomechanics. Clearly, we have much yet to learn about these demanding and existing forms of biathlon competitions.

## Author Contributions

H-CH initiated the study. ML, MJ, and H-CH contributed similarly to literature research and writing, approved the final version to be published, and agreed to be accountable for all aspects of the work.

## Conflict of Interest Statement

The authors declare that the research was conducted in the absence of any commercial or financial relationships that could be construed as a potential conflict of interest.

## Footnotes

1 Gear 2 (also referred to as V1 skate or offset skate, used primarily on moderate to steep terrain); Gear 3 (or V2 skate or double time, used mainly on level to moderate uphill terrain); Gear 4 (or V2 alternate skate or single time, used mostly on level terrain).
